# Endoplasmic reticulum facilitates the coordinated division of *Salmonella*-containing vacuoles

**DOI:** 10.1128/mbio.00114-25

**Published:** 2025-04-24

**Authors:** Umesh Chopra, Priyanka Bhansali, Subba Rao Gangi Setty, Dipshikha Chakravortty

**Affiliations:** 1 Department of Microbiology and Cell Biology, Indian Institute of Science, Bangalore, Karnataka, India; 2 School of Biology, Indian Institute of Science Education and Research Thiruvananthapuram, Thiruvananthapuram, Kerala, India; University of Florida College of Dentistry, Gainesville, Florida, USA

**Keywords:** *Salmonella*-containing vacuole, *Salmonella *effectors, ER tubules, ER contact sites

## Abstract

*Salmonella* Typhimurium (STM) resides in a membrane-bound compartment called the *Salmonella*-containing vacuole (SCV) in several infected cell types where bacterial and SCV division occur synchronously to maintain a single bacterium per vacuole. However, the mechanism behind this synchronous fission is not well understood. Fission of intracellular organelles is known to be regulated by the dynamic tubular endoplasmic reticulum (ER). In this study, we evaluated the role of ER in controlling SCV division. Interestingly, *Salmonella*-infected cells show activation of the unfolded protein response (UPR) and expansion of ER tubules. Altering the expression of ER morphology regulators, such as reticulon-4a (Rtn4a) and CLIMP63, significantly impacted bacterial proliferation, suggesting a potential role of tubular ER in facilitating SCV division. Live-cell imaging revealed the marking of tubular ER at the center of 78% of SCV division sites. This study also explored the role of SteA (a known *Salmonella* effector in modulating membrane dynamics) in coordinating the SCV division. SteA resides on the SCV membranes and helps form membrane contact between SCV and ER. The colocalization of ER with SCV enclosing STMΔ*steA* was significantly reduced, compared with SCV of STM WT or STMΔ*steA:steA*. STMΔ*steA* shows profound defects in SCV division, resulting in multiple bacteria in a single vacuole with proliferation defects. *In vivo*, the STMΔ*steA* shows a defect in colonization in the spleen and liver and affects the initial survival rate of mice. Overall, this study suggests a coordinated role of bacterial effector SteA in promoting ER contact/association with SCVs and regulating SCV division.

**IMPORTANCE:**

This study highlights the essential role of the host endoplasmic reticulum in facilitating SCV division and maintaining a single bacterium per vacuole. The *Salmonella* effector SteA helps maintain the single bacterium per vacuole state. In the absence of SteA, *Salmonella* resides as multiple bacteria within a single large vacuole. The STMΔ*steA* shows reduced proliferation under *in vitro* conditions and exhibits colonization defects *in vivo*, highlighting the importance of this effector in *Salmonella* pathogenesis. These findings suggest that targeting SteA could provide a novel therapeutic approach to inhibit *Salmonella* pathogenicity.

## INTRODUCTION

*Salmonella enterica* subsp. *enterica* serovar Typhimurium (STM) is an intracellular pathogen known to infect a wide range of hosts and result in diseases across species, including gastroenteritis and systemic infections (1, 2). *S*. Typhimurium enters the body through the fecal-oral route, following which the bacteria utilize an arsenal of strategies to overcome several stresses like the acidic pH of the stomach, gut flora, bile salts, etc., and finally reach the small intestinal epithelia (3). *Salmonella* secretes several virulence proteins inside the cell, which leads to actin rearrangement and membrane ruffling, which helps in the invasion of bacteria inside the host epithelial cell (4–7). *Salmonella* effector SopD is known to facilitate the recruitment of dynamin2 (Dyn2) at the invasion site, which helps in the endocytosis of bacteria inside host cells (8).

Upon invasion into the host cell, *Salmonella* resides in the *Salmonella*-containing vacuole (SCV), a membrane-enclosed compartment. Mature SCV shares several characteristic markers with lysosomes/late endosomes, such as LAMP1 and Rab7, but is negative for acid hydrolases and cathepsins (9). Inside the epithelial cells, several protruding structures from SCV known as *Salmonella*-induced filaments (SIFs) are observed, which are LAMP1 positive (10, 11). The membrane composition of SCV is not very well understood, but it contains molecules and proteins from several host organelles and is enriched with several bacterial effector proteins (12, 13). Our group, as well as several others, has previously shown that *Salmonella* resides as a single bacterium per vacuole state inside the host, suggesting that the SCV membrane divides synchronously with the division of *Salmonella,* but the mechanism behind SCV division is not known (14–17). For successful SCV division, expansion of SCV membrane, pinching of SCV membrane precisely with bacterial division, and finally, scission of host-derived SCV membrane must be followed in an orchestrated manner.

Several publications have highlighted the importance of contact sites between cellular organelles, such as contact sites of ER with mitochondria, plasma membrane, and endosomes and contact sites between mitochondria with lysosomes (18–21). Contact sites between ER-mitochondria and ER-endosomes are very widely studied in the aspect of organelle division (18, 22). In the case of mitochondria, which comprises an inner and an outer membrane, both membranes divide simultaneously during its fission. The inner mitochondrial membrane uses its machinery for fission, whereas outer membrane constriction is initiated by the ER, followed by the recruitment of Drp1, which mediates the fission of mitochondria (18, 23, 24). Bacterial pathogens like *Chlamydia* and *Brucella* make contact sites with cellular organelles to facilitate their proliferation inside the host cell (25–27). Interestingly, the SCV membrane also comprises several ER proteins, and recently, membrane contact sites between the SCV and ER have also been observed (28). To understand the mechanism behind the SCV division, we reasoned that SCV is partially like mitochondria. SCV and mitochondria (being an endosymbiont) encompass an outer membrane derived from the eukaryotic host, whereas the inner membrane of mitochondria is prokaryotic in nature, like the cell wall of *Salmonella*. To understand the same, we hypothesized that a process similar to mitochondrial fission could lead to SCV division. Our results suggest that *Salmonella* infection leads to the activation of UPR, ER expansion, and an increase in ER tubulation. We have observed ER tubules marked at the fission site of the SCV division, possibly initiating the constriction. *Salmonella* translocated effector SteA localizes onto the SCV membrane and possibly helps in making contact with ER, suggesting that SteA can act as a factor to recruit the ER onto SCV and thus facilitate the SCV division. STMΔ*steA* resides as multiple bacteria in one big vacuole with defects in proliferation. During *in vivo* infection, STMΔ*steA* shows defects in colonization in the spleen and liver if injected through the intraperitoneal route. Also, there is a delay in the onset of mice death upon infection with STMΔ*steA* compared with STM WT, but eventually, mice succumbed to death 8 days post-infection. Overall, our study unravels, for the first time, the coordinated role of ER and bacterial effector SteA in successful SCV division inside the cell.

## MATERIALS AND METHODS

### Bacterial strains and culture conditions

*Salmonella enterica* subspecies *enterica* serovar Typhimurium (STM WT) wild-type strain ATCC 14,028 s was used in all experiments. The bacterial strain was cultured in Luria broth (LB-Himedia) with constant shaking (170 rpm) at 37°C orbital shaker. Antibiotics like kanamycin (50 µg/mL) and ampicillin (50 µg/mL) were used wherever required. A one-step gene inactivation method was used for the generation of knockout strains (29). Wild-type as well as mutant strains were transformed with pFPV-m-cherry or pFPV-GFP plasmid for immunofluorescence assays. A list of bacterial strains used in this study is added in Table S1.

### Generation of complement strain and SteA-eGFP plasmid

Low copy number plasmid pQE-60 was used to prepare the SteA-HA complement strain. Briefly, PCR amplification (using Thermo Q5 DNA polymerase) of the *steA* gene was performed using a wild-type STM colony with respective PCR primers. Forward cloning primer was chosen from a position of 250 base pairs upstream of the gene start site to amplify the native promoter of the gene during PCR and to maintain the native expression level of protein, and the sequence of HA tag was added in reverse primer. The amplified PCR product was purified using a gel extraction kit (Qiagen). The PCR product and plasmid were subjected to restriction digestion by using Xho1 (NEB) and Hind III (NEB) at 37°C for 2 h. The double-digested PCR product and plasmid were further subjected to ligation using T4 DNA ligase (NEB) at 16°C for 12–14 h. The ligated product was further electroporated in the STMΔ*steA* strain to prepare the complemented strain, that is, STMΔ*steA*:pQE60-*steA* (hence referred to as STMΔ*steA:steA*). The expression level of complement was confirmed with western blotting and confirmed with DNA sequencing.

For the preparation of SteA-eGFP plasmid for transfection study, an eGFP-N3 empty vector was used. The sequence of *steA* was amplified via PCR from STM WT using primers with restriction sites for Xho1 and BamH1. The entire process for cloning was performed as discussed above.

### Eukaryotic cell culture, transfection, and plasmids

HeLa cells and RAW 264.7 murine macrophages were cultured in Dulbecco's modified Eagle medium (DMEM) (Lonza) containing 10% fetal bovine serum (FBS) (Gibco) at a humified incubator, maintaining a temperature of 37°C and 5% CO_2_. Cells were seeded in 6-well, 12-well, or 24-well plates at a confluency of 60%–70% prior to infection. Cells were maintained in the presence of 1% penicillin and streptomycin (penstrep). Cells were given a phosphate buffered saline (PBS) wash and supplemented with fresh media without penstrep for at least 6 h prior to infection. Plasmid overexpression or knockdown via shRNA was done using Lipofectamine 3000 using the manufacturer’s protocol. Briefly, 300 ng of plasmid was mixed in opti-MEM media for 5 min, whereas 1 µL of L3000 was added (for one well of 24-well plates) in opti-MEM for 5 min separately and further mixed and incubated for 20 min at room temperature. Post-incubation, this mixture was added to cells for 6 h for transfection to take place. Cells were washed with PBS and supplemented with complete media post 6 h of treatment, and post 48 h of transfection, infection was given. Overexpression constructs, such as pHAGE2 mCherry-Rtn4a, were a kind gift from Prof. Tom Rapoport (Addgene plasmid 86683) (30); mCh Climp-63 and mCh sec61β were a kind gift from Prof. Gia Voeltz (Addgene plasmid 136293, Addgene 49155) (31, 32), and RFP KDEL construct was a kind gift from Prof. Nagaraj Balasubramanian (33). GFP-LAMP1 was provided by Prof. Mahak Sharma (34). The shRNA plasmids were obtained from the human genome-wide TRC shRNA library (purchased from Sigma-Aldrich, USA, Catalog no. SH0411), available at shRNA Resource Centre, MCB, IISc. pLKO.1-puro non-mammalian shRNA (SHC002, shControl) was used as a control in all the shRNA-mediated knockdown experiments. Plasmid sequences and details are added in Table S2.

### Protocol for infection (gentamicin protection assay)

The cells were seeded at the required confluency and infected with *Salmonella* strains with actively growing log phase culture (for epithelial cells) and stationary phase culture (for RAW 264.7 macrophages) at a multiplicity of infection (MOI) of 10 for intracellular survival assay (ICSA) and confocal imaging. For the live cell imaging experiment, an MOI of 25 was used to increase the event of finding infected cells for imaging. For confocal microscopy studies, cells were seeded on sterile glass coverslips at least 24 h before infection. Upon infecting the cells at the required MOI, the plate was centrifuged at 600 rpm for 5 min to facilitate the adhesion, and then, the plate was incubated for 25 min at 37°C and 5% CO_2_. Post-incubation, bacteria-containing medium was removed, and cells were given two PBS washes to remove any extracellular bacteria. Fresh medium containing 100 µg/mL of gentamicin was added into wells and incubated at 37°C for 1 h. Following this, cells were again given two PBS washes, and fresh medium containing 25 µg/mL of gentamicin was added, and this concentration was maintained till the end point of the experiment. Cells were harvested at required time points, such as 2 h and 16 h for ICSA and 2 h, 6 h, and 16 h for confocal microscopy-based experiments.

### Confocal microscopy

After an appropriate hour post-infection, cells were washed with PBS twice and fixed with 3.5% PFA at room temperature for 15 min. After fixation, coverslips were washed with PBS, and cells were stained with primary antibody prepared in 2% bovine serum albumin (BSA) in PBS carrying 0.01% of saponin (for membrane permeabilization) for 1 h at room temperature or 4°C overnight in a cold room as required. Post-incubation, coverslips were washed again twice with PBS and incubated with specific secondary fluorophore-labeled antibodies and incubated for 1 h at room temperature. Coverslips were then mounted on clean glass slides using mounting media. Once the mounting media was dried, coverslips were stabilized by sealing the periphery with transparent nail paint on the sides. Images were acquired on a confocal scanning laser microscope (Zeiss 880 multiphoton microscope or Leica SP8 Falcon system with or without zoom as required). Zen black software was used for all colocalization-based quantification and to calculate the mean fluorescence intensity (MFI). Calculations of cell total corrected fluorescence (CTCF), percent of ER tubules, SCV diameter, and line scans were prepared using ImageJ. The ER tubule percentage is calculated by calculating the sheet percentage from the total cell area: sheet area/total area × 100 = sheet area percentage, and further 100 − sheet area percentage = tubule area percentage (35). Live cell microscopy was performed on the Leica SP8 Falcon microscope, and live cell video was processed and prepared using LAS X software. Antibodies used in this study are mentioned in Table S3.

### Bacterial enumeration after infection for intracellular survival assay

After the appropriate hour of post-infection, cells were washed with PBS at RT and lysed using 0.1% Triton-X 100. Cell lysate was plated on SS agar to obtain colony-forming unit (CFU), and it was used for the calculation of percent invasion and fold proliferation using this formula:


$$\begin{array}{l} Percent\;invasion=(CFU\;obtained\;at\;2\;hours\;post\text{-}infection/CFU\;obtained\;from\;pre\text{-}inoculum)\times 100\\ Fold\;proliferation=CFU\;obtained\;at\;16\;hours\;post\text{-}infection/CFU\;obtained\;at\;2\;hours\;post\;infection \end{array}$$

### RNA isolation and real-time PCR (qRT-PCR)

RNA isolation was performed using TRIzol (Takara) following the manufacturer’s protocol. Quantification of RNA was done using nano-drop (Thermo-Fisher Scientific), and RNA samples were run on 1% agarose gel to check the quality of RNA. In addition, 2 µg of RNA was subjected to DNase I (Thermo Fisher Scientific) treatment at 37°C for 1 h followed by the addition of 0.5M EDTA (final concentration 5 mM) and heat inactivation at 65°C for 10 min to inactivate the enzyme. Furthermore, cDNA was prepared by using a cDNA synthesis kit (Takara) as per the manufacturer’s instructions. The expression profile of all genes of interest was evaluated using qRT primers by using SYBR green mix (Takara) in a Biorad Real-Time PCR machine. The expression value of the target gene was normalized to housekeeping internal control β-actin and further compared with uninfected cells. Primers used in this study are mentioned in Table S4.

### *In vivo* animal experiment

In total, 6- to 8-week-old C57BL/6 female mice were infected by orally gavaging 10^7^ CFU of STM WT, STMΔ*steA,* and STMΔ*steA:steA*. Five days post-infection, mice were euthanized, and organs such as liver, spleen, MLN, and blood were harvested to study the colonization in organs. For intraperitoneal injection, 10^3^ CFU of bacteria was administered per animal, and 3 days post-infection, organs were homogenized and further plated on *Salmonella-Shigella* agar. Obtained CFU values were normalized with the gram weight of the organ and then converted into a log scale. Blood was collected through heart puncture and plated on SS agar. For the mice survival assay, 10^8^ CFU was administered to each mouse via oral gavage. Mice were monitored every day until the time none of the mice in the cohort survived.

### Statistical analysis

Statistical analysis was performed using GraphPad Prism software. For analysis, the Student’s *t*-test (unpaired parametric test) or two-way analysis of variance (ANOVA) was performed. The results are indicative of mean ± SD or mean ± SEM as mentioned in figure legends. To calculate the statistical significance of bacterial colonization *in vivo*, the Mann-Whitney test was performed. Group size, experimental number, number of cells in each set, and *P* value of each experiment are described in figure legends.

## RESULTS

### *Salmonella* infection leads to the activation of UPR and expansion of the endoplasmic reticulum

**Fig 1:**
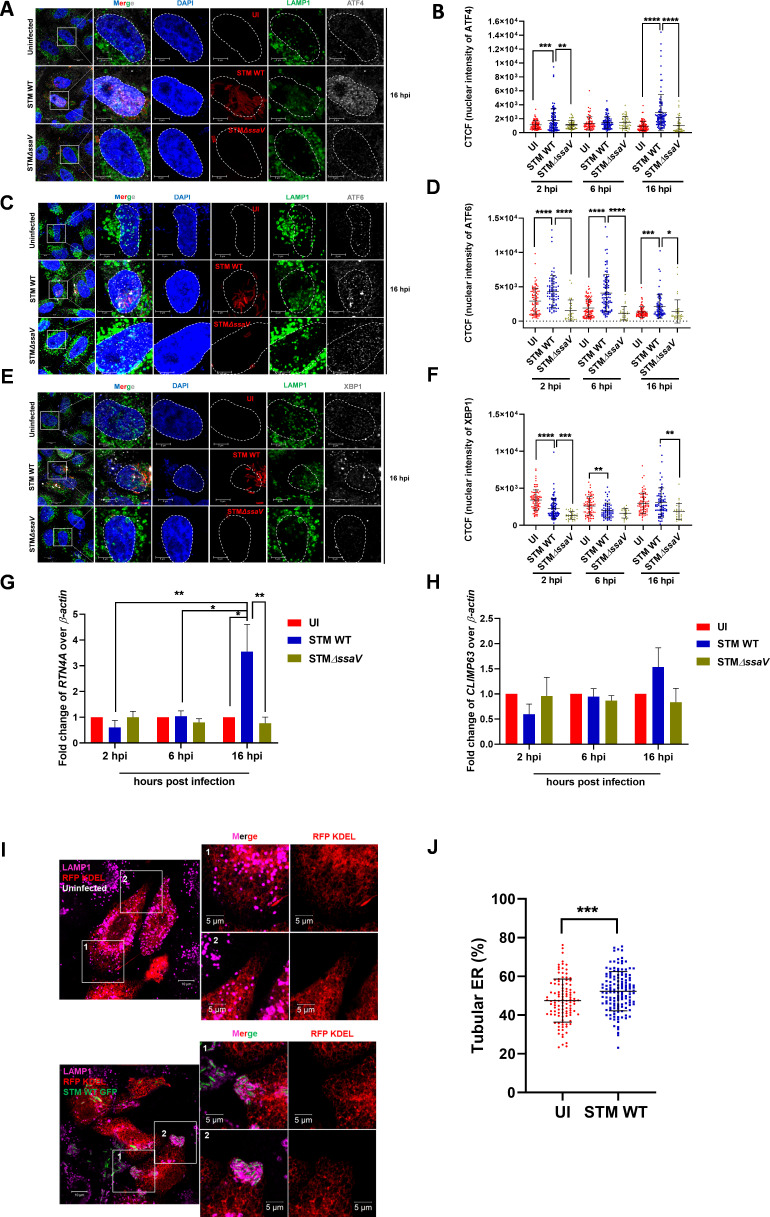
*Salmonella* infection leads to the activation of UPR and expansion of the endoplasmic reticulum. (**A**) Representative confocal microscopy images of uninfected and infected HeLa cells with STM WT mCh or STMΔ*ssaV* mCh at MOI 10, 16 h post-infection and immuno-stained for ATF4, LAMP1, and DAPI. (**B**) Represents the quantification of CTCF intensity of ATF4 inside the nucleus; data are representative of *n* = 100–120 cells, from four independent experiments, mean ± SD. (**C**) Representative confocal microscopy images of uninfected and infected HeLa cells with STM WT mCh or STMΔ*ssaV* mCh at MOI 10, 16 h post-infection and immuno-stained for ATF6, LAMP1, and DAPI. (**D**) Represents the quantification of cell total corrected fluorescence (CTCF) intensity of ATF6 inside the nucleus; data are representative of *n* = 60–90 cells from three independent experiments, mean ± SD (**E**) Representative confocal microscopy images of uninfected and infected HeLa cells with STM WT mCh or STMΔ*ssaV* mCh at MOI 10, 16 h post-infection and immuno-stained for XBP1, LAMP1, and DAPI. (**F**) Represents the quantification of CTCF intensity of XBP1 inside the nucleus; data are representative of *n* = 60–90 cells from three independent experiments, mean ± SD. (**G**) Quantitative real-time PCR of *RTN4a* (**H**) and *CLIMP63*, *N* = 3, *n* = 3, mean ± SEM. (**I**) Confocal microscopy images of uninfected and infected HeLa cells showing ER expansion. (**J**) Represents the quantification of tubular ER, data is representative of *n* = 100–150 cells from four independent experiments, mean ± SD. A two-way ANOVA test was used to analyze the data in panels G and H. Student’s *t*-test (unpaired) was used to analyze the data in panels B, D, F, and J. A white dotted line in panels A, C, and E represents the boundary of the nucleus, and hpi represents the hours post-infection. ****P < 0.0001, ***P < 0.001, **P < 0.01, *P < 0.05.

Several bacterial and viral infections are known to activate UPR for their survival (36, 37). UPR helps alleviate ER stress by initiating several transcriptional and translational regulations that lead to the maintenance of ER homeostasis (38, 39). Inside the cell, UPR signaling is maintained by transmembrane proteins located in the ER membrane and their catalytic domain toward the cytosol. Inositol-requiring enzyme-1 (IRE-1), protein kinase-c like ER kinase (PERK), and activating transcription factor-6 (ATF-6) comprise the main arm of UPR. Recent reports have shown the contact of SCV with ER, and the presence of ER proteins in the membrane fraction of SCV, suggests the crosstalk between them (12, 28). Here, we investigated how *Salmonella* infection modulates the ER dynamics. To understand the activation of UPR by any of these pathways, we used confocal microscopy and quantified the translocation of downstream transcription factors, such as XBP1, ATF4, and ATF6, inside the nucleus upon infection. SPI2 mutants of *Salmonella*, STMΔ*ssaV* (which is unable to secrete SPI-2 encoded effector proteins in host cytosol), were kept as a control to understand if activation of UPR is mediated by bacterial effectors or dependent on bacterial invasion. Our results suggest that from as early as post 2 h of *Salmonella* infection, both the transcription factors, ATF4 and ATF6, from the cytosol translocated to the nucleus of infected cells compared with uninfected cells and STMΔ*ssaV* infected cells, suggesting that only wild-type *Salmonella* can activate UPR (Fig. 1A through D). *Salmonella-*induced ATF4 and ATF6 translocation were retained until 16 h post-infection (Fig. S1A through D). However, we did not observe any increase in XBP1 recruitment inside the nucleus (Fig. 1E and F) upon infection at any time point post-infection (Fig. S1E and F), suggesting that *Salmonella* infection upregulates UPR by PERK and ATF6 pathways in HeLa cells. To alleviate the ER stress, activation of UPR leads to the expansion of the ER, protein folding capacity, and increased phospholipid production (40). Increased bacterial proliferation requires a continuous source of membrane for the increasing number of SCV, and increased phospholipid production upon UPR could facilitate the SCV proliferation inside the host cell. Therefore, we sought to determine if *Salmonella* infection led to ER expansion during the pathogenesis phase. Our transcriptional analysis suggests that upon infection, there is an upregulation at the transcript level of ER shaping proteins such as *Reticulon-4a* (*RTN4A*, essential for maintaining ER tubules) (Fig. 1G). The upregulation was significantly observed at the late time point of infection, that is, 16 h post-infection, suggesting that active proliferation of *Salmonella* and not just invasion (as evident at 2 h and 6 h post-infection) leads to ER expansion. We also observed an overall increase in the transcript levels of *CLIMP63* (essential for maintaining ER sheets) as well 16 h post-infection, but it was not statistically significant (Fig. 1H). We further quantified the levels of ER-resident proteins Calnexin and BiP during infection. Consistent with the transcript data, we observed an increase in the level of Calnexin upon infection with STM WT at 16 h post-infection compared with STMΔ*ssaV* (Fig. S1G and H). In contrast, the level of BiP showed a reduction at the initial time points but remained unchanged at the late time point post-infection (Fig. S1I and J).

Since an increase in the transcript levels of ER shaping proteins was observed at the later time of infection, we tried to quantify the percentage of ER sheets and tubules using the ER marker, RFP-KDEL, upon infection to understand ER expansion. Upon analysis of microscopy images, we observed that *Salmonella*-infected cells showed an increased percentage of ER tubules compared with uninfected cells (Fig. 1I and J). The changes in the percentage of ER tubules were only evident in infected cells compared with uninfected and bystander cells (neighboring cells that are not infected), suggesting that stabilized SCV and bacterial effectors are responsible for this phenotype (Fig. S1K and L). Together, these data indicate that *Salmonella* infection leads to changes in ER morphology.

### Expansion of ER tubules facilitates SCV proliferation and its division

**Fig 2:**
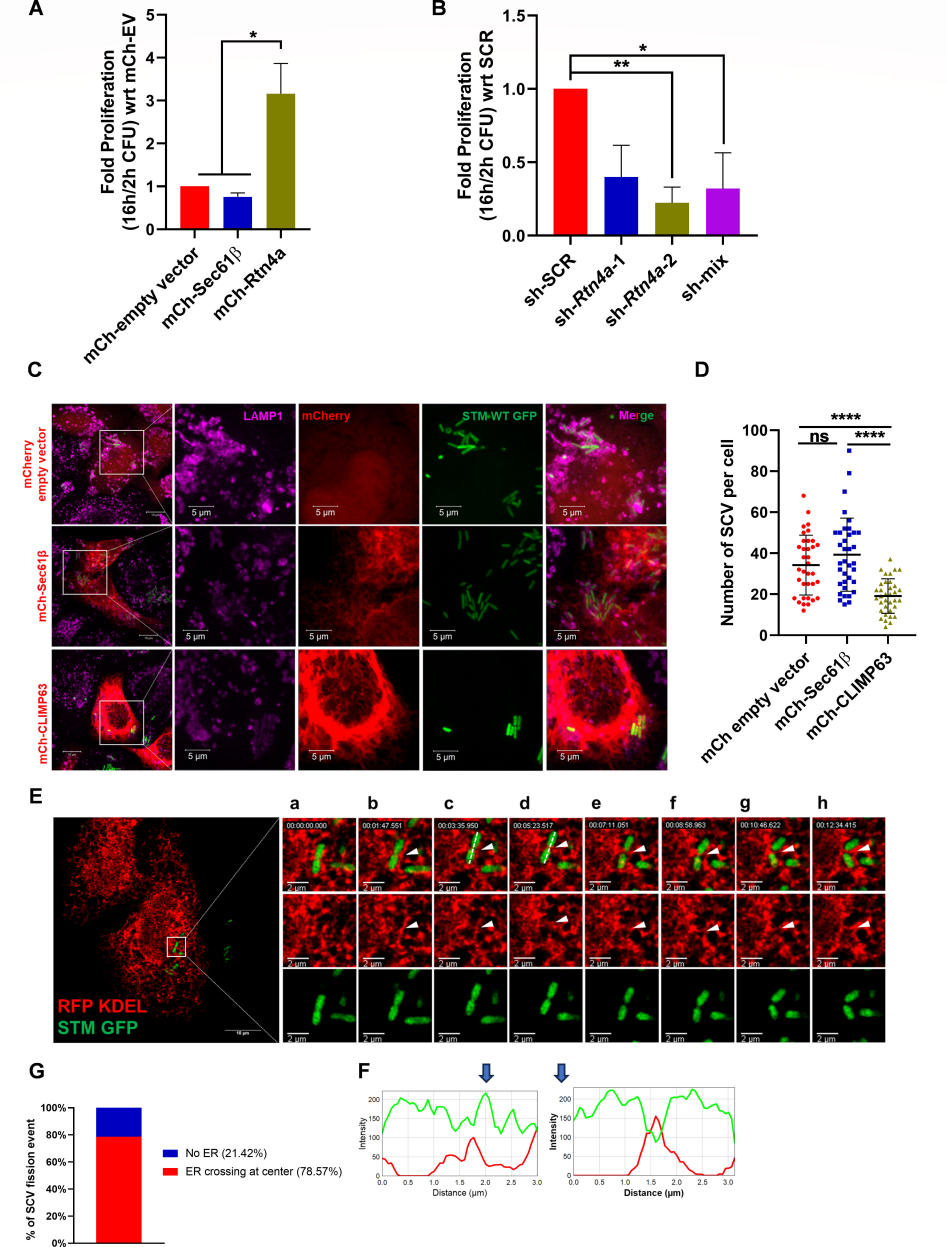
Expansion of ER tubules facilitates SCV proliferation and its division. (**A**) Represents the ICSA upon overexpression of Rtn4a; data are representative of *N* = 3, *n* = 3 mean ± SEM. (**B**) ICSA upon Rtn4a knockdown in HeLa cells; data are representative of *N* = 3, *n* = 3 mean ± SEM. (**C**) Representative confocal microscopy images of SCV number per cell upon overexpression of mCh-EV, mCh-Sec61β, and mCh-CLIMP63, 16 h post-infection. (**D**) Quantification of the number of SCV per cell, 16 h post-infection; data are representative of *n* = 40–60 cells from two independent experiments. (**E**) Snapshot of live cell imaging of *Salmonella* infected HeLa cells from Movie S1, White arrowhead represents the ER overlap at dividing bacteria. A white dotted line along the length of bacteria represents the axis used for the line scan. Inset from a to h represents the time snapshot of live cell imaging. (**F**) Shows the line scan of images. Blue arrows represent the snapshot for which the line scan is analyzed (snapshots c and d). (**G**) Quantification of SCV fission events marked by ER; data are representative of *n* = 14 SCV fission events from four independent experiments. Student’s *t*-test was used to analyze the data. ****P < 0.0001, ***P < 0.001, **P < 0.01, *P < 0.05.

Dynamic ER tubules play an essential role in forming membrane contact sites with several organelles (41, 42). Figure 1 indicates that actively proliferating bacteria increased tubular ER and upregulated the expression of *RTN4A*. We also tried to understand if there is an advantage of the increased tubules to bacterial proliferation. To identify the role of ER tubules in mediating bacterial proliferation, we transiently overexpressed reticulon 4 a (Rtn4a) in HeLa cells, which helps increase ER tubules (43) and then quantified the proliferation of bacteria by intracellular survival assay (ICSA). Compared with empty vector and general ER marker mCh-sec61β control, increased ER tubulation by Rtn4a led to higher proliferation of *Salmonella* with no significant change in percent invasion, suggesting that an increase in ER tubules helps in bacterial proliferation (Fig. 2A; Fig. S2A). To validate if this change in proliferation was dependent solely on the function of Rtn4a, the knockdown of *RTN4A* via shRNA was performed (Fig. S2B). Knockdown of Rtn4a (approximately 40% reduction at transcript level) led to reduced proliferation of bacteria compared with scrambled control, thereby strengthening the crucial role of ER tubules in bacterial proliferation (Fig. 2B). Additionally, we performed confocal microscopy to quantify the number of SCVs per cell upon overexpression of Rtn4a. The data indicate an increase in SCV numbers per cell at 16 h post-infection compared with the empty vector control and the Sec61β control (Fig. S2C and D). CLIMP63 overexpression led to an increase in ER sheets as opposed to tubules. We quantified the number of SCVs per cell upon transiently overexpressing Climp63 in HeLa cells. Our data indicated that the number of SCVs present per cell decreases upon overexpressing ER sheets using CLIMP63 compared with controls (Fig. 2C and D). Similarly, overexpression of CLIMP63 led to reduced proliferation of *Salmonella* compared with control (Fig. S2E). The division of *Salmonella* is linked with the fission of SCV membrane (15). Since the intricate balance of ER sheets and tubules affects bacterial proliferation, we hypothesized that ER tubules might facilitate SCV fission. In the case of mitochondria, membrane-less organelles, and endosomes, ER marks the division site, and later, fission is completed by Drp1 (in mitochondria) and coronin1c and TMCC 1 complex (in endosome) (18, 24, 44, 45). To investigate our hypothesis of ER tubules playing a role in SCV fission, we performed live cell imaging in cells transfected by RFP-KDEL (to mark the ER), followed by infection with *Salmonella*. Quantification of data suggests that ER tubules mark the division site precisely at the center of dividing SCV in 78% of the events, as observed by image snapshots and corresponding line scans (Fig. 2E through G; Movie S1). STM WT-infected RAW macrophages also showed contact with ER on the dividing bacteria (Movie S2; Fig. S2F). This was also evident in fixed HeLa cell images where we could observe Lamp1-enclosed *Salmonella* division in SCV marked by ER tubules exactly in the center in 56.7% ± 6.61% of fission events; however, in 14% ± 9.07% of the events, ER was seen in proximity to the center and hence mentioned as adjacent, whereas 29.2% ± 8.4% of dividing SCV did not possess ER tubules on them (Fig. S2G and H). These data suggest the importance of ER tubules in marking the fission site of SCV. Taken together, these data suggest that ER tubules help in the proliferation and division of SCV by marking the fission site inside the cell and helping in maintaining SCV number within the cell.

### *Salmonella* translocated effector SteA is crucial for maintaining the association of SCV with ER, resulting in a single bacterium per vacuole

**Fig 3:**
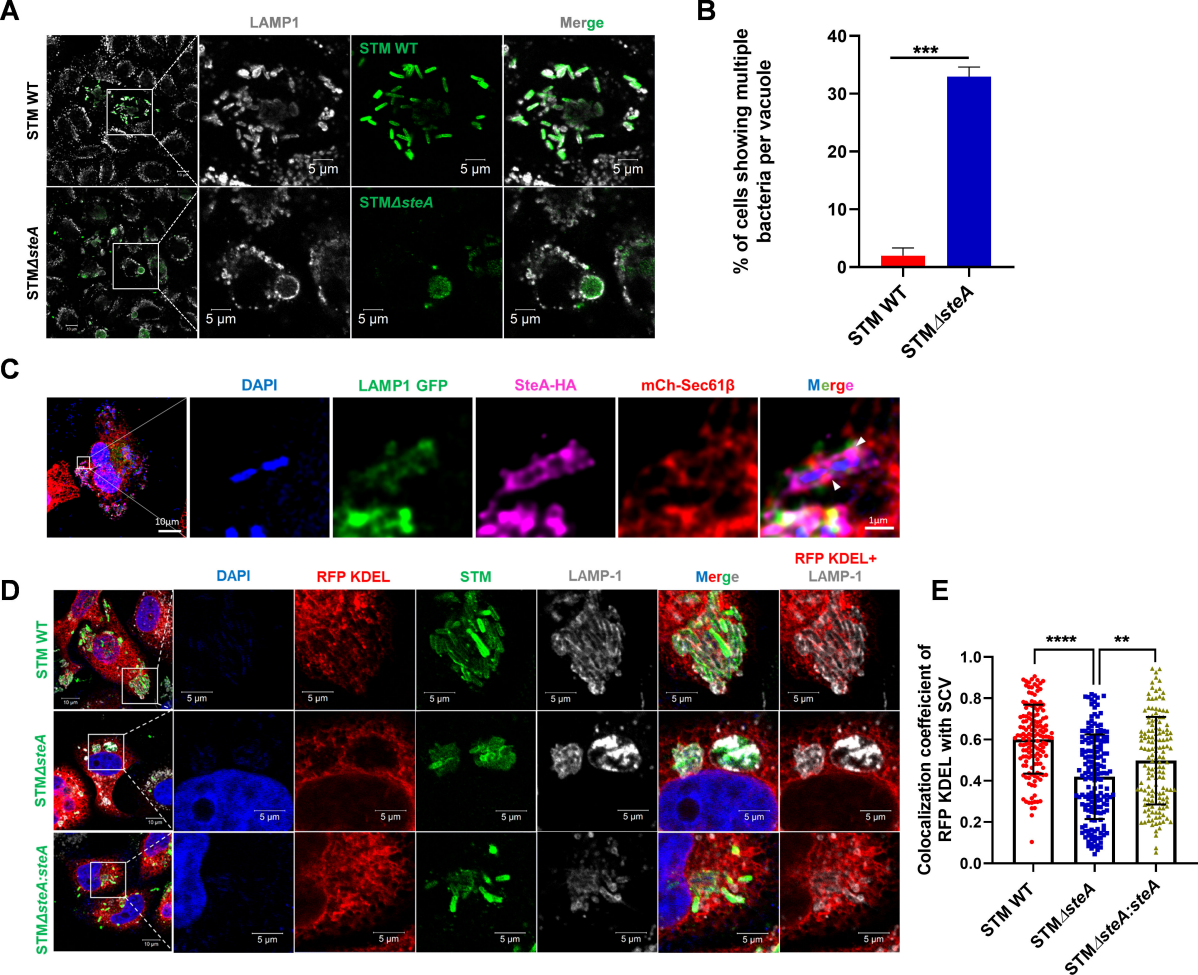
*Salmonella* translocated effector SteA is crucial for maintaining the association of SCV with ER, resulting in a single bacterium per vacuole. (**A**) Represents confocal microscopy images showing SCV formed by STM WT and STMΔ*steA,* 16 h post-infection. (**B**) Quantification of the percentage of cells showing multiple bacteria in one vacuole, 16 h post-infection; data are representative of *n* = 120–150 cells from three independent experiments, mean ± SEM. (**C**) Representative confocal image of infected HeLa cell showing contact between SteA-HA and ER; arrowheads represent the interaction between ER and SteA-HA. (**D**) Microscopy images represent the colocalization of ER with SCV formed by STM WT, STMΔ*steA*, or STMΔ*steA:steA,* 16 h post-infection. (**E**) Quantification of colocalization coefficient of ER marker RFP KDEL with SCV marker LAMP1. Data are representative of *n* = 60–80 cells from three independent experiments, 16 h post-infection, mean ± SD. Student’s *t*-test was used to analyze the data. ****P < 0.0001, ***P < 0.001, **P < 0.01, *P < 0.05.

SCV division leads to a single bacterium per vacuole throughout the course of pathogenesis in cell lines or *in vivo* (14). Domingues et al. suggested the role of SteA in maintaining the membrane dynamics of SCV (46). In accordance with the same, we also observed that the *steA* mutant strain of *Salmonella* (STMΔ*steA*) resides as multiple bacteria in one single vacuole (Fig. 3A). Our data suggest that compared with STM WT, 30% ± 3% of mutant bacteria reside in one big vacuole, suggesting that the loss of function of *steA* leads to improper SCV fission (Fig. 3B). Live cell imaging shows several points of contact between SCV and ER, suggesting that SCV actively associates with ER for its survival and proliferation (Fig. 2E and 3C). SteA is known to reside on the PI4P-enriched SCV membrane (Fig. 3C), and hence, we asked if membrane contacts between ER and SCV are maintained by SteA (47). Microscopy images show that SteA-enriched SCV makes contact with ER (Fig. 3C). Further quantification of the data suggests that ER colocalizes more with the SCV of STM WT and STMΔ*steA:steA* (complement strain) than the SCV formed by STMΔ*steA* (as observed by colocalization between SCV marker, Lamp1, and ER marker, RFP KDEL), suggesting that SteA helps in making contacts of SCV with ER (Fig. 3D and E). This was also observed upon transfecting HeLa cells with the SteA-eGFP construct, where SteA-eGFP showed higher colocalization with ER marker RFP-KDEL compared with eGFP empty vector control, strengthening the fact that SteA possibly associated with ER (Fig. S3A and B). Overall, these data suggest that SteA might help in making contact/association of SCV with ER and further recruiting ER onto the SCV. These contacts can be used as a stealthy approach by *Salmonella* to recruit membrane from ER, along with other *Salmonella* effector proteins, facilitating SCV fission.

### STMΔ*steA* shows a defect in proliferation and pathogenicity *in vivo*

**Fig 4:**
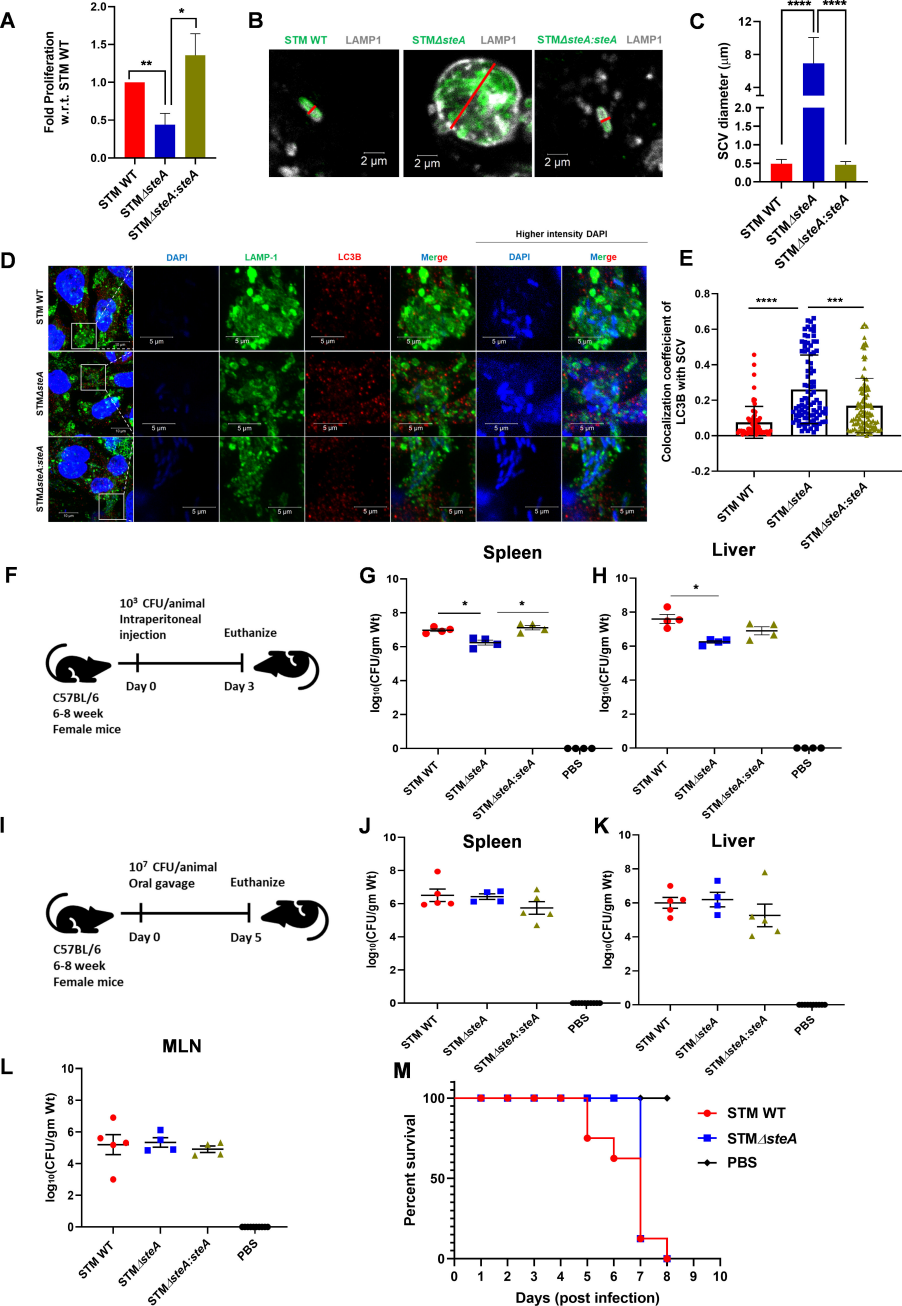
STMΔ*steA* shows a defect in proliferation and pathogenicity *in vivo.* (**A**) Intracellular survival assay of STM WT, STMΔ*steA*, and STMΔ*steA:steA* in HeLa cells; data are representative of *N* = 3, *n* = 3, mean ± SEM. (**B**) Line representation to quantify SCV diameter where the red line indicates the length of SCV used for quantification. (**C**) Quantification of SCV diameter; data are representative of 100–150 cells from three independent experiments, 16 h post-infection, mean ± SD. (**D**) Representative microscopy images showing colocalization of LC3B with SCV, 16 h post-infection. High intensity of DAPI represents the *Salmonella* in SCV. (**E**) Quantification of colocalization coefficient of LC3B with SCV; data are representative of *n* = 80–100 cells from three independent experiments, mean ± SD. (**F**) Outline of experimental procedure of mice infection through intraperitoneal route. (**G**) CFU enumeration in spleen and (**H**) liver; data are representative of *N* = 2, *n* = 5 mice per cohort. (**I**) Outline of experimental procedure of mice infection through oral gavage and dissection on day 5 post-infection. (**J**) CFU enumeration in organs like spleen, (**K**) liver, (**L**) and MLN; data are representative of *N* = 3, *n* = 5 mice per cohort, mean ± SD. (**M**) Survival curve of the cohort of mice infected with STM WT and STM*ΔsteA, n* = 8 mice per cohort. Mann-Whitney test was performed to obtain statistical significance for *in vivo* CFU data. Student’s *t*-test was used to analyze the data from panels A through E. ****P < 0.0001, ***P < 0.001, **P < 0.01, *P < 0.05.

Previous reports from our lab suggested that *Salmonella* utilizes the strategy of SCV division to increase the number of SCV per cell; hence, it becomes difficult for the cellular system to eradicate the growing SCV (14). Since *steA* mutants form a big vacuole inside the cell, we asked how the mutant strain proliferates in the cell. Compared with STM WT and STMΔ*steA:steA* (complement strain), STMΔ*steA* proliferated significantly less in the HeLa cell line (Fig. 4A). The reduced proliferation of the mutant strain suggests the possibility that those bulky vacuoles might be targeted by cellular machinery for degradation. Quantification of vacuole size suggests that the diameter of SCV formed by STMΔ*steA* is around 6.9 ± 3.13 µm, whereas SCV of STM WT is 0.4 ± 0.11 µm and SCV of STMΔ*steA:steA* is 0.46 ± 0.13 (Fig. 4B and C). To explore the possibility of whether the bulky vacuoles are targeted by the host autophagy machinery, we investigated the colocalization of the autophagy marker LC3B with SCVs. Quantification of the data revealed that LC3B colocalized more frequently with SCVs of *steA* mutants compared with SCVs of STM WT or STMΔ*steA*:steA at 16 h post-infection (Fig. 4D and E). Actively proliferating *Salmonella* also reduces the number of lysosomes inside the cell to escape its fusion with acidic lysosomes (14). In cells infected with STMΔ*steA*, we observed a higher intensity of Lamp1, comparable with that in uninfected cells. This suggests that STMΔ*steA* is unable to alter lysosomal dynamics, unlike STM WT, and increased flux of lysosomal proteins, along with the recruitment of LC3B, may contribute to the reduced proliferation of STMΔ*steA* within host cells (Fig. S4A and B). Next, we were interested in understanding the pathogenicity of STMΔ*steA in vivo*. For this, we intraperitoneally injected 10^3^ CFU of *Salmonella* per C57BL/6 mice, and 3 days post-infection, mice were euthanized, and organs such as liver and spleen were harvested to observe organ burden. Our data suggest that 3 days post-infection, STMΔ*steA* shows less colonization in the spleen and liver compared with STM WT and STMΔ*steA:steA* (Fig. 4G and H). To further understand the pathogenicity of the mutant strain, we orally gavaged C57BL/6 mice with 10^7^ CFU of STM WT, STMΔ*steA*, and STMΔ*steA:steA,* and 5 days post-infection, mice were euthanized, and organs such as liver, spleen, and mesenteric lymph nodes were harvested since they act as a secondary site of infection (Fig. 4J through L). We observed no significant change in the colonization of bacteria in these organs, suggesting that STMΔ*steA* does not lose its capability to cross the gut barrier and colonize and disseminate in distal organs and blood if injected through the oral route (Fig. S4C). However, upon performing a survival assay of mice upon infection through oral gavage, we noticed the delayed onset of mice death in the STMΔ*steA* infected cohort (Fig. 4M), where STM WT infected mice started succumbing to infection from 5 days post-infection, STMΔ*steA* infected cohort started succumbing to infection at 7 days post-infection, and at 8 days post-infection, all the mice succumbed to death. Taken together, these data suggest that if the fission of SCV is altered, *Salmonella* resides as multiple bacteria in one big vacuole and proliferates less. Those big/bulky vacuoles are further targeted by autophagic machinery and cleared by active lysosomes *in vitro*. During *in vivo* infection through the intraperitoneal route, STMΔ*steA* shows defects in colonization in the spleen and liver and also affects the initial survival rate of mice, suggesting that SteA acts as an essential effector for pathogenicity in mice.

## DISCUSSION

Bacterial pathogens invade several cell types and reside in endocytic or phagocytic vacuoles (48). Cells use their extraordinary machinery to clear the pathogens, but by using several effector proteins, bacterial pathogens modulate cellular machinery and prolong their survival. A few strategies that several bacterial pathogens utilize are modification of the membrane of the pathogen-containing vacuole, blocking the activation of xenophagy, and escaping its fusion with the acidic lysosome (49–55). Bacterial pathogens such as *Legionella, Brucella, Chlamydia*, and *Salmonella* are known to reside in vacuoles, but the morphology and membrane source of the vacuole are quite different. *Legionella* and *Chlamydia* reside in a spacious vacuole that comprises multiple bacteria in a single vacuole (56), whereas *Salmonella* resides in a tight-fitting vacuole as a single bacterium per vacuole (14). The biogenesis of the SCV membrane is not very well known, but proteome analysis of SCV suggests that the SCV membrane is highly modified and contains proteins from the lysosome, Golgi, and ER (12). Several effectors of *Salmonella* help in recruiting membrane components, inhibiting the transport of M6PR, and making contact sites with organelles for its proliferation (57–59). Our results suggest that *Salmonella* infection induces ER stress inside the cell and activates the UPR. To alleviate the ER stress, UPR leads to the expansion of ER inside the cell. Our data suggest that wild-type STM infection leads to UPR activation, whereas STMΔ*ssaV* is unable to activate the UPR arm. This suggests that bacterial effectors are crucial for initiating ER stress, as it was evident as early as 2 h post-infection. Our studies support the previous findings, which suggest that *Salmonella-*induced ER stress is crucial for bacterial proliferation (60–62). Furthermore, UPR activation leads to an increase in phospholipid production (40), which could be useful for the bacteria as growing SCV requires a continuous source of membrane. *Salmonella-*infected cells show an increase in expression of *RTN4A* and *CLIMP63* with an increase in tubular ER, suggesting that this is an active strategy employed by bacteria for survival. Exogenous overexpression of Rtn4a provides an advantage in the proliferation of *Salmonella*; however, reduction in ER tubules by knockdown of Rtn4a or overexpression of ER sheets using CLIMP63 negatively affects the bacterial proliferation and SCV number inside the cell, indicating ER tubules play a crucial role in bacterial proliferation. The intricate balance of ER sheets and tubules has been recently shown to affect the dynamics of membrane-less organelles (42). Our study also highlights the importance of ER in the successful proliferation of *Salmonella*. Our live cell data showed the recruitment of ER tubules at the center of dividing SCV. This was evident in HeLa as well as RAW 264.7 macrophage cell lines, suggesting that ER tubule recruitment marks the division site of SCV and leads to a single bacterium per vacuole. ER is very well known for marking the division site of organelles such as mitochondria, membrane-less organelles, and endosomes (18, 42). Our study suggests that *Salmonella* hijacks the strategy of ER recruitment for its survival and SCV division. We have identified the role of bacterial effector SteA in maintaining the contact/association of SCV with ER. Earlier studies have shown the presence of SteA on SCV and its colocalization with Golgi (47, 63, 64). In our study, we have partially observed a similar phenotype and have observed a new role of SteA in interaction with ER. Inside the infected cell, the expression of SteA is highly regulated as it can be mostly seen around the bacteria onto SCV. To investigate the role of SteA in making contact with ER, we looked for the colocalization of ER with SCV of STM WT and STMΔ*steA*. We observed the difference in colocalization of ER with SCV in the absence of SteA, highlighting the importance of the *Salmonella* effector SteA in mediating the contacts between SCV and ER. Bacterial pathogens such as *Chlamydia* use their effector protein for forming contacts of their inclusion bodies with ER (25), and they also target host cellular protein to facilitate this interaction (65). *Salmonella* effectors SseJ and SseL have previously been reported to recruit OSBP1 onto SCV, which helps in making contact with VapA and VapB (conserved ER proteins known for tethering of ER with cellular organelles) and maintaining SCV vacuolar integrity (66). Our data suggest the potential role of SteA in associating the SCV with ER as a strategy to facilitate its proliferation.

The loss of function of SteA led to a bulky vacuole carrying multiple bacteria inside and with defects in proliferation. Evidence from McQuate et al. also suggests the defect in the proliferation of STMΔ*steA* in epithelial cells as well as macrophages (63). To understand the difference in the proliferation of STMΔ*steA* compared with STM WT, we have observed that SCVs formed by STMΔ*steA* colocalize more with the autophagy marker LC3B, suggesting that big bulky vacuoles are cleared by the cellular system and hence lead to less proliferation inside the cell. STM WT also actively reduces lysosome number inside the cell as an active strategy to block its fusion with the acidic lysosome (14). However, in the case of STMΔ*steA*-infected cells, we observed a higher mean intensity of LAMP1, which was similar to the intensity observed in uninfected cells, suggesting that somehow STMΔ*steA* could not modulate lysosomal dynamics, and as a result, active lysosomes could hinder bacterial proliferation in the cell line model. Similarly, Liu et al. have highlighted the importance of SteA in bacterial proliferation inside cells by impairing the fusion of SCV with lysosomes (67). Taken together, these data suggest the importance of SteA in bacterial proliferation inside infected cells. STMΔ*steA* also showed defects in colonization in the spleen and liver if infected through the intraperitoneal route, suggesting that SteA is crucial for *in vivo* pathogenicity. However, to our surprise, we did not observe any defect in the *in vivo* colonization of mouse organs such as the liver, spleen, MLN, and blood if infected through the oral gavage route. Survival analysis of mice suggests late onset of mice death in STMΔ*steA* infected cohort upon infection compared with STM WT. We have also observed less weight reduction in STMΔ*steA* infected mice cohort compared with STM WT, and this difference in weight reduction could provide an advantage in the initial survival rate of STMΔ*steA* infected mice (Fig. S4D). Geddes et al. have reported the importance of SteA for efficient colonization in mouse spleen using the BALB/c mice model infecting via the intraperitoneal route (64). We have also observed similar defects in the spleen using C57BL/6 mice. However, upon infection through oral gavage, there are no defects in the colonization of STMΔ*steA* in organs. This observation suggests that the route of infection can severely affect bacterial colonization and pathogenicity. Infection through the intraperitoneal route bypasses the gut-intestinal barrier, although during oral gavage, *Salmonella* faces several challenges, from the acidic pH of the stomach to several metabolites in the intestinal lumen. More studies are needed to understand this difference in colonization rate depending on the route of infection. Alongside, further investigations are needed to understand the vacuolar status of SCV during *in vivo* infection if infected through the oral or intraperitoneal route to understand the difference in pathogenicity. Taken together, our study suggests the importance of SteA in bacterial pathogenicity and proliferation.

Our study provides new insights into how bacteria use a strategy of remodeling the ER dynamics for its benefit; Domingues et al. and Chen et al. have highlighted the importance of SteA for the active division of SCV (46, 68). Our data support their findings and suggest the possible role of SteA in recruiting the ER and facilitating SCV fission. Interestingly, wild-type STM can easily divide along with SCV, where loss of function of SteA hampers 30% SCV division with marked less colocalization with ER, suggesting that there can be several other effector proteins that might facilitate the contacts between ER and SCV. As reported by Chen et al. (68) about the coordinated roles of SteA, SopD2, and SifA in successful SCV division, our data explain the newly identified role of SteA in the process by associating with ER. Since these effectors have coordinated roles, it will be interesting to understand how individual bacterial effectors are crucial in the complete fission of SCV. Several host proteins have also been linked with SCV division, as the absence of those host proteins, such as PLEKHM1 and ESCRT-3, leads to SCV carrying multiple bacteria (58, 69). Within the cell, SCV acts as an autonomous organelle that has acquired a vacuolar membrane from the host system, which also synchronously divides along with bacterial division. The machinery regulating the SCV division could be difficult to identify because these fission events are very transient in nature. As per literature evidence, both host and bacterial effectors could be equally involved in this process to execute successful fission. A complete knowledge of fission machinery can be used as a targeted approach to inhibit bacterial proliferation during infection.

## SUPPLEMENTAL MATERIAL

Fig. S1A to J mbio.00114-25-s0001.tif *Salmonella* infection leads to the activation of UPR and expansion of the endoplasmic reticulum.

Fig. S1K and L mbio.00114-25-s0002.tif *Salmonella* infection leads to the activation of UPR and expansion of the endoplasmic reticulum.

Fig. S2 mbio.00114-25-s0003.tif Expansion of ER tubules facilitates SCV proliferation and its division.

Fig. S3 mbio.00114-25-s0004.tif *Salmonella* translocated effector SteA is crucial for maintaining the association of SCV with ER, resulting in a single bacterium per vacuole.

Fig. S4 mbio.00114-25-s0005.tif STMΔ*steA* shows a defect in proliferation and pathogenicity *in vivo*.

Graphical Abstract mbio.00114-25-s0006.tif Visual diagram of study highlights.

Supplemental Legends mbio.00114-25-s0007.docx Legends for supplemental figures and movies.

Movie S1 mbio.00114-25-s0008.avi Expansion of ER tubules facilitates SCV proliferation and its division (related to Fig. 2E).

Movie S2 mbio.00114-25-s0009.avi Expansion of ER tubules facilitates SCV proliferation and its division (related to Fig. S2F).

ASM does not own the copyrights to Supplemental Material that may be linked to, or accessed through, an article. The authors have granted ASM a non-exclusive, world-wide license to publish the Supplemental Material files. Please contact the corresponding author directly for reuse.
