# Supplementary figures and images for: Endoplasmic reticulum facilitates the coordinated division of Salmonella-containing vacuoles
Source: mBio. 2025 Apr 24;16(5):e00114-25. doi: 10.1128/mbio.00114-25 (PMC12077215; doi:10.1128/mbio.00114-25)

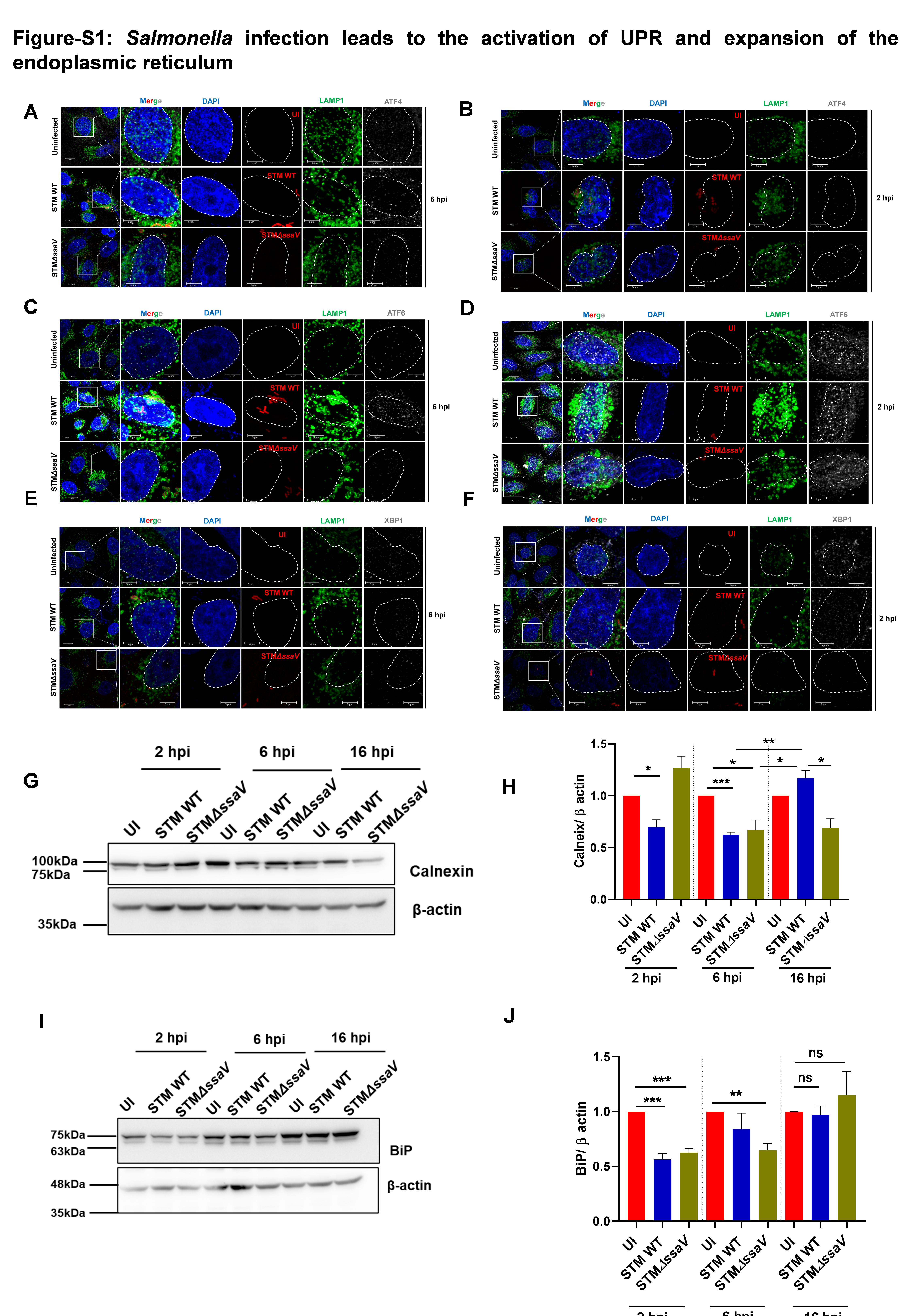

Supplement: Fig. S1A to J — Salmonella infection leads to the activation of UPR and expansion of the endoplasmic reticulum. [file mbio.00114-25-s0001.tif]

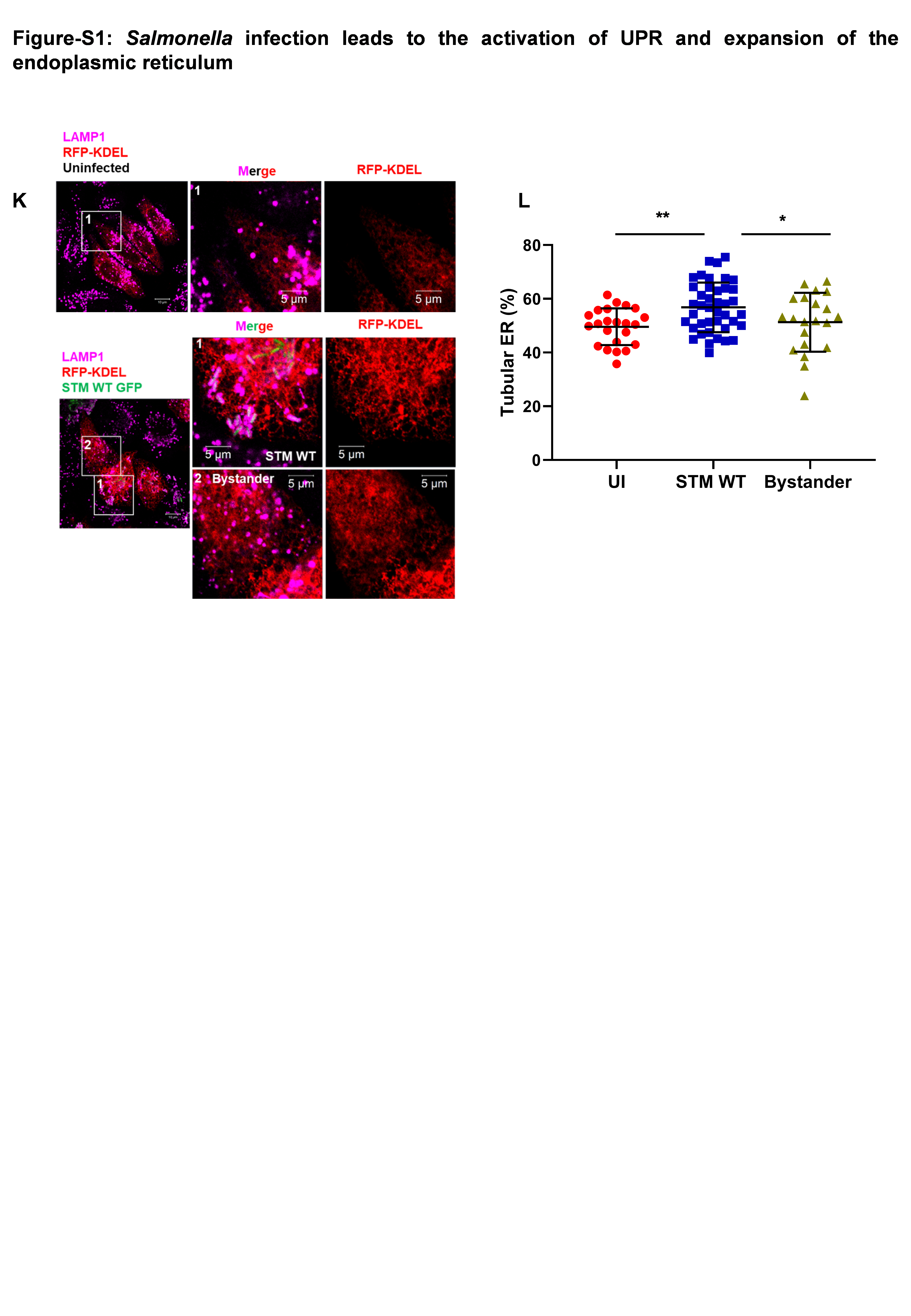

Supplement: Fig. S1K and L — Salmonella infection leads to the activation of UPR and expansion of the endoplasmic reticulum. [file mbio.00114-25-s0002.tif]

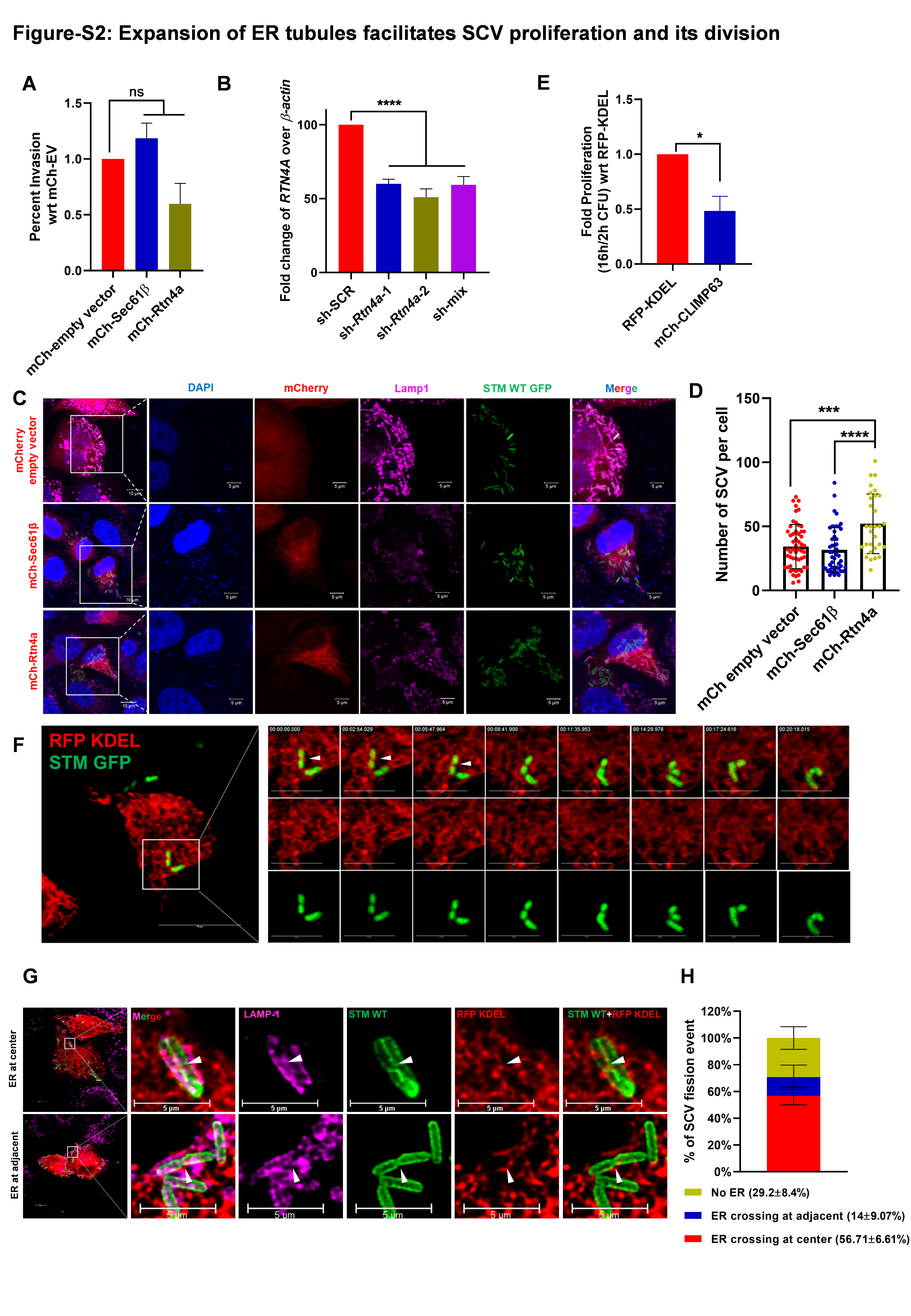

Supplement: Fig. S2 — Expansion of ER tubules facilitates SCV proliferation and its division. [file mbio.00114-25-s0003.tif]

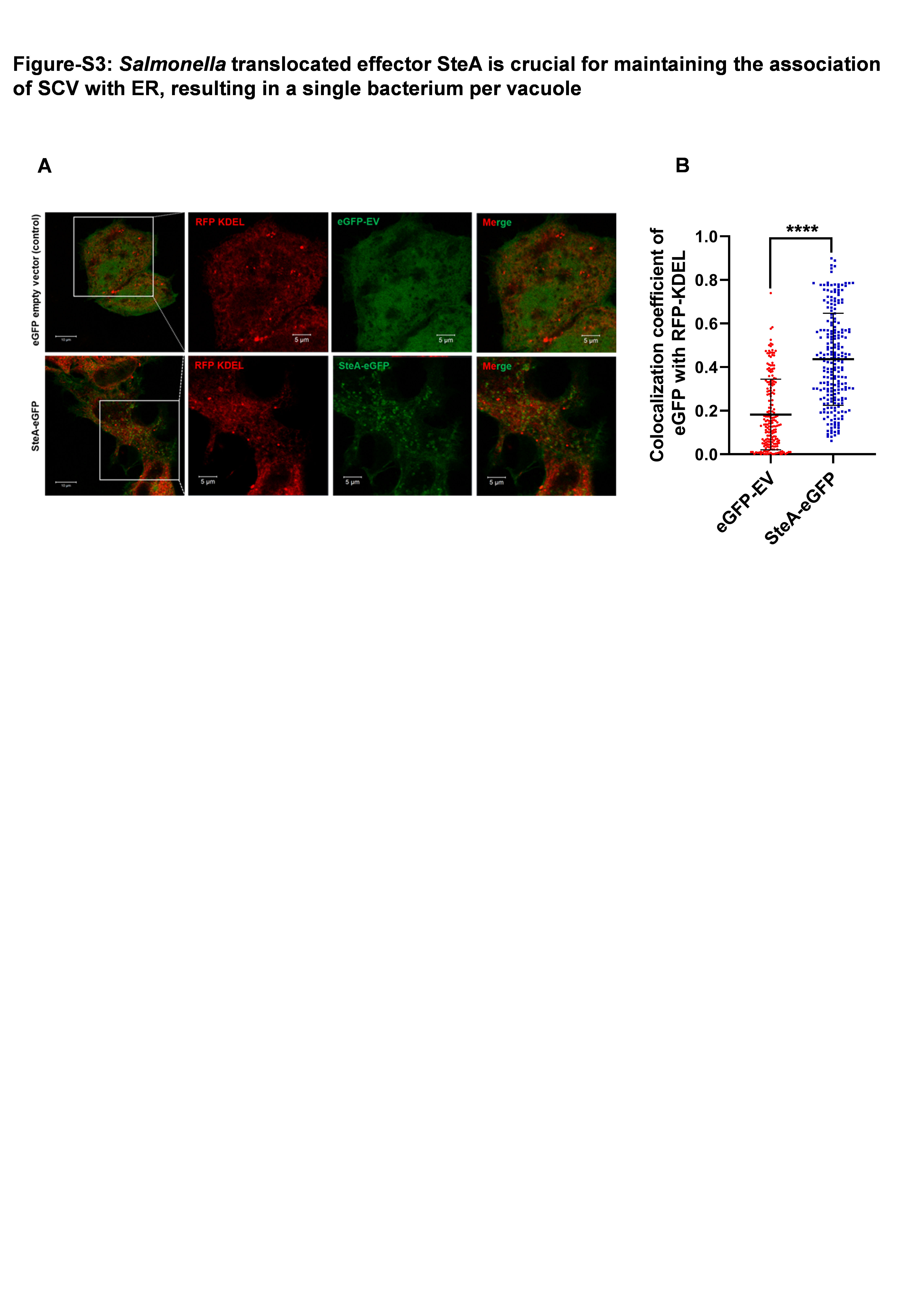

Supplement: Fig. S3 — Salmonella translocated effector SteA is crucial for maintaining the association of SCV with ER, resulting in a single bacterium per vacuole. [file mbio.00114-25-s0004.tif]

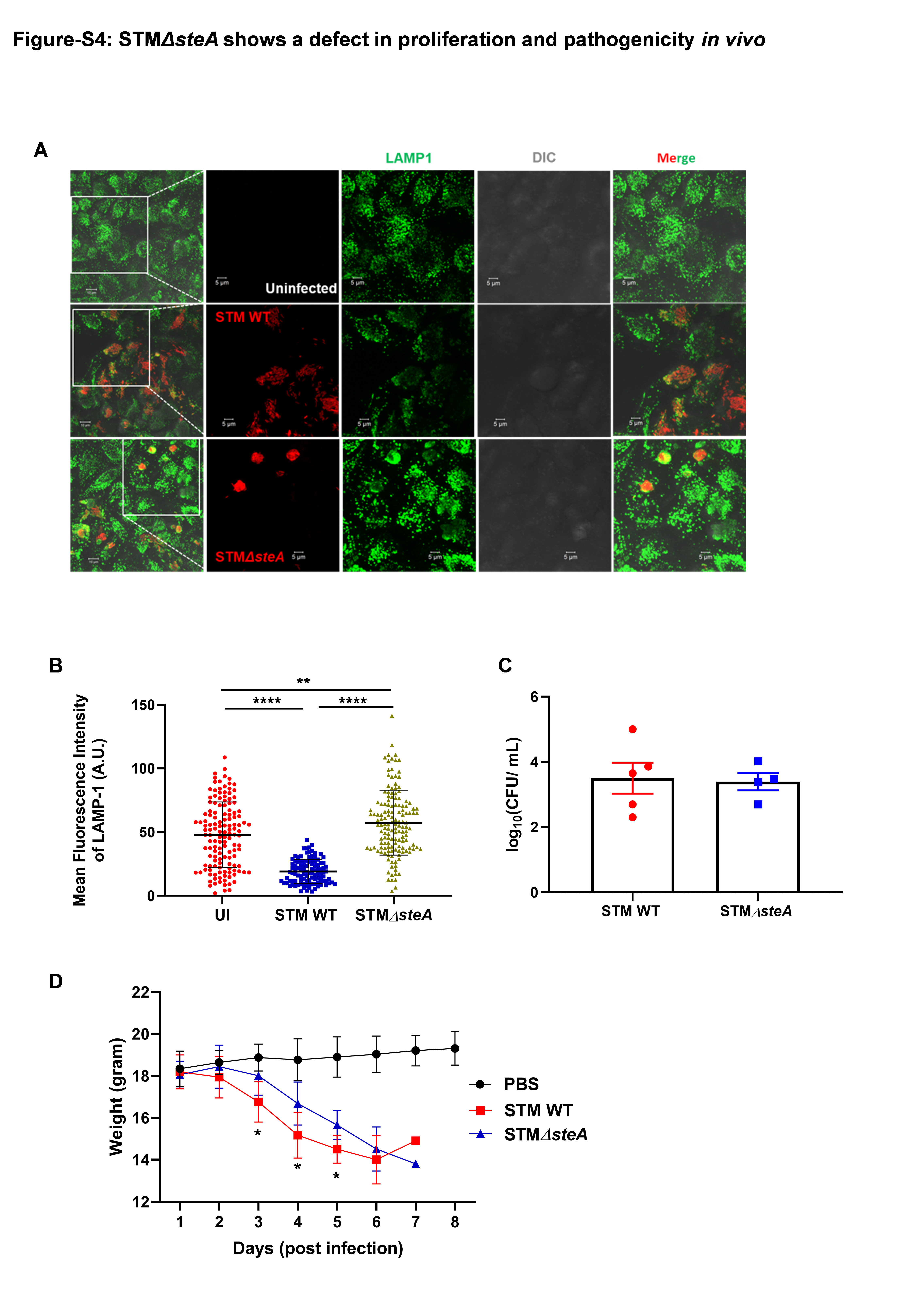

Supplement: Fig. S4 — STMΔsteA shows a defect in proliferation and pathogenicity in vivo. [file mbio.00114-25-s0005.tif]

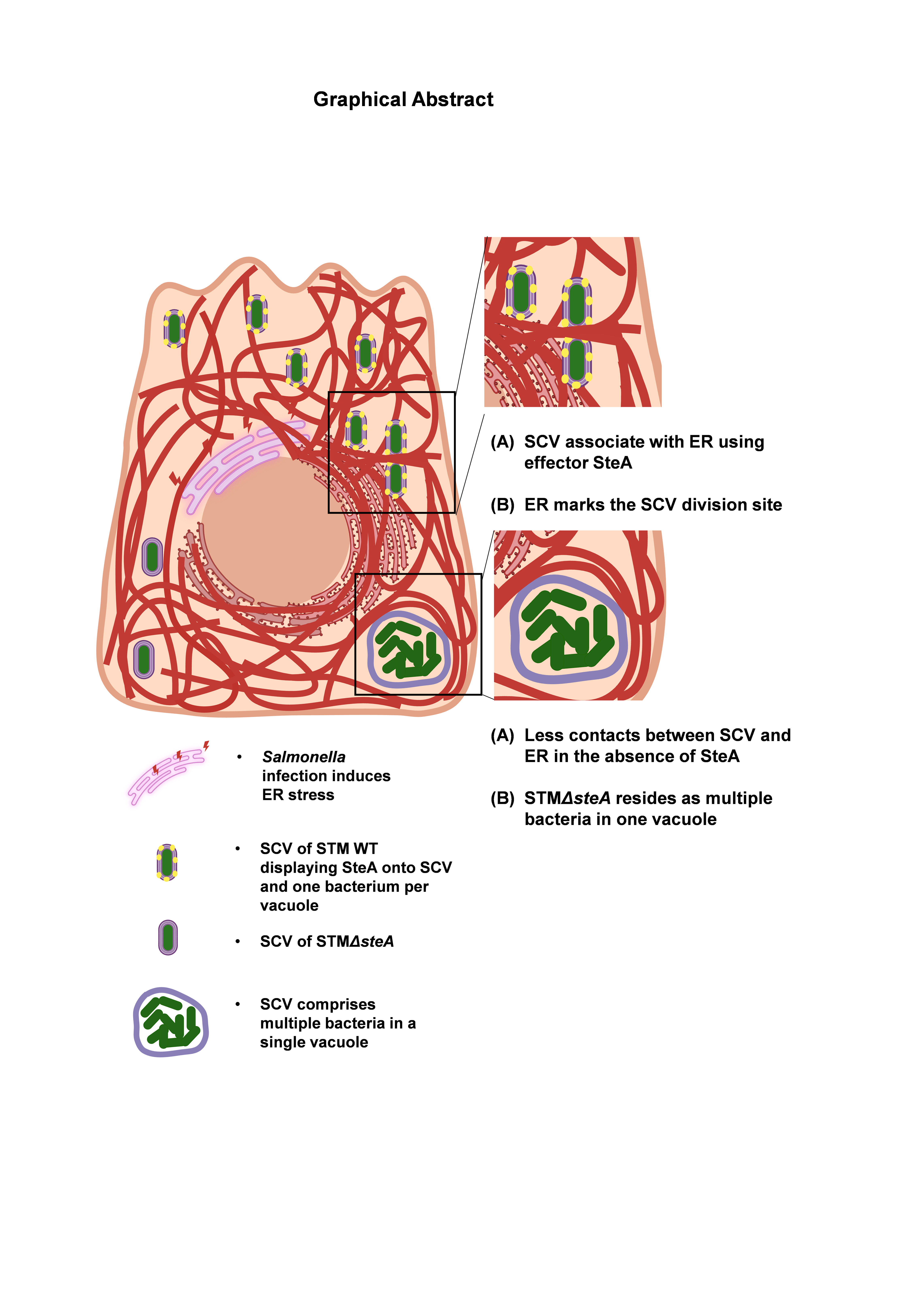

Supplement: Graphical Abstract — Visual diagram of study highlights. [file mbio.00114-25-s0006.tif]
