# Supplementary material for: Endoplasmic reticulum facilitates the coordinated division of Salmonella-containing vacuoles
Source: mBio. 2025 Apr 24;16(5):e00114-25. doi: 10.1128/mbio.00114-25 (PMC12077215; doi:10.1128/mbio.00114-25)
Supplement: Supplemental Legends — Legends for supplemental figures and movies. [file mbio.00114-25-s0007.docx]

**Endoplasmic reticulum facilitates the coordinated division of *Salmonella* containing vacuoles**

Umesh Chopra^1^, Priyanka Bhansali^1^, Subba Rao Gangi Setty^1^, Dipshikha Chakravortty^1,2*^

^1^Department of Microbiology and Cell Biology, Indian Institute of Science, Bangalore, Karnataka 560012, India

^2^School of Biology, Indian Institute of Science Education and Research Thiruvananthapuram, Maruthamala PO, Vithura, Thiruvananthapuram - 695551, Kerala, India

^*^Corresponding Author- dipa@iisc.ac.in

**Legends to Supplementary Figures and Movie**

**Figure-S1: *Salmonella* infection leads to the activation of UPR and expansion of the endoplasmic reticulum**

Representative confocal microscopy images of uninfected, STM WT mCh and STM*ΔssaV* mCh infected cells, immuno-stained for (A) ATF4, LAMP1, and DAPI, 6 hours post-infection (B) ATF4, LAMP1, and DAPI, 2 hours post-infection (C) ATF6, LAMP1 and DAPI, 6 hours post-infection (D) ATF6, LAMP1 and DAPI, 2 hours post-infection (E) XBP1, LAMP1 and DAPI, 6 hours post-infection (F) XBP1, LAMP1 and DAPI, 2 hours post-infection (G) Immunoblotting of Calnexin upon infection of HeLa cells with STM WT or STM*ΔssaV* at 2, 6, and 16 hours of post-infection (H) Quantification of band intensities of Calnexin normalized to β actin (loading control). (I) Immunoblotting of BiP upon infection of HeLa cells with STM WT or STM*ΔssaV* at 2, 6, and 16 hours of postinfection (J) Quantification of band intensities of BiP normalized to β actin (loading control) (K) Representative confocal microscopy images of uninfected, STM WT infected, and bystander cells, showing an expansion of ER tubules, 16 hours post-infection (L) Quantification of ER tubules, data is representative of n=20-40 cells from 2 independent experiments, mean±SD. Student’s t-test was used to analyze the data. ****<0.0001, ***<0.001, **<0.01, *<0.05.

**Figure-S2: Expansion of ER tubules facilitates SCV proliferation and its division**

(A) Percent invasion of STM WT in HeLa cells upon overexpressing mCh-EV, mCh-Sec61β, and mCh-Rtn4a (B) Quantitative real time PCR of *RTN4A* upon knockdown, Data is representative of N=3, n=3, mean±SD (C) Representative confocal microscopy images of SCV number per cell upon overexpression of mCh-EV, mCh-Sec61β, and mCh-Rtn4a, 16 hours post infection (D) Quantification of the number of SCV per cell, 16 hours post infection, data is representative of n=50-60 cells from 2 independent experiments (E) Represents the ICSA of *Salmonella* WT upon overexpression of CLIMP63 and RFP KDEL (control), data is representative of N=3, n=3 mean± SEM (F) Live cell imaging snapshots of RAW 264.7 murine macrophage cell line infected with STM WT showing ER overlap at fission site, N=1, (Movie S2) (G) Confocal microscopy images representing SCV fission events marked by ER, at the center or adjacent (H) Quantification of fission events marked by ER, data is representative of n=49 SCV fission events from 4 independent experiments. The white arrowhead represents the ER overlap at dividing SCV. Student’s t-test was used to analyze the data ****<0.0001, ***<0.001, **<0.01, *<0.05.

**Figure-S3: *Salmonella* translocated effector SteA is crucial for maintaining the association of SCV with ER, resulting in a single bacterium per vacuole**

(A) Representative microscopy images showing colocalization of eGFP or SteA-eGFP with RFP KDEL (ER marker) (B) Quantification of colocalization coefficient of eGFP or SteA-eGFP with RFP KDEL. Data is representative of n=80-100 cells from 2 independent experiments, mean±SD. Student’s t-test was used to analyze the data ****<0.0001, ***<0.001, **<0.01, *<0.05.

**Figure-S4: STM*ΔsteA* shows a defect in proliferation and pathogenicity *in vivo***

(A) Representative images of uninfected and infected HeLa cells with STM WT and STM*ΔsteA* showing the intensity of LAMP1 (B) Quantification of mean fluorescence intensity of LAMP1, data is representative of n=100-150 cells from 3 independent experiments, mean±SD (C) CFU enumeration of STM WT and STM*ΔsteA* in the blood of C57BL/6 mice, 5 days post oral gavage, data is representative of N=3, n=5 mice per cohort, mean±SD (D) Quantification of weight reduction in mice upon infection with STM WT and STM*ΔsteA* till 8 days post-infection, n=8 mice per cohort*.* Mann-Whitney test was performed to calculate statistical significance in Fig. S4C. Student’s t-test was used to analyze the data in Fig. S4B and S4D. ****<0.0001, ***<0.001, **<0.01, *<0.05**.**

**Legends to movie**

**Movie S1: Expansion of ER tubules facilitates SCV proliferation and its division**

Time lapse movie corresponding to Fig. 2E showing the ER tubules (red) labelled with RFP-KDEL and STM WT (green- expressing GFP) in HeLa cells.

**Movie S2: Expansion of ER tubules facilitates SCV proliferation and its division**

Time lapse movie corresponding to Fig. S2F showing the ER tubules (red) labelled with RFP-KDEL and STM WT (green-expressing GFP) in RAW 264.7 macrophage.

**Supplementary file: Table:1 List of bacterial strains used in this study**

| Strain | Characteristic (Antibiotic resistance) | Source |
| --- | --- | --- |
| STM WT 14028s | - | Kind gift by Prof. M. Hensel |
| STM*ΔssaV* | Kan^R^ | Laboratory stock |
| STM*ΔsteA* | Kan^R^ | This study |
| STM*ΔsteA:steA* | Kan^R^ +Amp^R^ | This study |

**Supplementary File: Table: 2 List of plasmids used for overexpression.**

| Plasmid | Source |
| --- | --- |
| 1. pHAGE2 mCherry-Rtn4a (Plasmid-86683) | pHAGE2 mCherry-Rtn4a was a gift from Tom Rapoport (Addgene plasmid # 86683) |
| 1. mCh-Climp63 (Plasmid #136293) | mCh-Climp63 was a gift from Gia Voeltz (Addgene plasmid # 136293) |
| 1. RFP-KDEL | RFP KDEL construct was a kind gift from Prof. Nagaraj Balasubramanian |
| 1. LAMP1-GFP | GFP-LAMP1 construct was provided by Prof. Mahak Sharma |
| 1. mCh-Sec61β | mCh sec61β was a kind gift from Prof. Gia Voeltz (Addgene 49155) |

**List of shRNA plasmids used for knockdown**

| **S.No.** | **Target Gene** | **TRC ID** | **Sequence** |
| --- | --- | --- | --- |
| 1 | RTN4 reticulon 4 [ *Homo sapiens* (human) ] | TRCN0000179649 | CCGGGCAGTGTTGATGTGGGTATTTCTCGAGAAATACCCACATCAACACTGCTTTTTTG |
| 2 | RTN4 reticulon 4 [ *Homo sapiens* (human) ] | TRCN0000147624 | CCGGGCATATCTGGAATCTGAAGTTCTCGAGAACTTCAGATTCCAGATATGCTTTTTTG |

**Supplementary File: Table:3 List of Antibodies**

| **Antibody** | **Catalog number** |
| --- | --- |
| ATF-4 | CST-11815s |
| ATF-6 | ATF-6α Antibody (H-280): sc-22799 |
| XBP-1 | XBP-1 Antibody (M-186): sc-716 |
| LAMP1 | DSHB 1D4B |
| Anti-Salmonella | Invitrogen- **PA1-20811** |
| LC3B | Sigma-L7543 |
| BiP | CST-3177 |
| Calnexin | CST-2679 |

**Supplementary File: Table-4, List of Primers used in this study:**

| **S.No** | **Primer Name** | **Sequence (5’-3’)** |
| --- | --- | --- |
| 1 | steA-KO-FP | CAAATAGTTATGGTAGCGAGCTTTTATGTCGGCCGCCCATCATATGAATATCCTCCTTAG |
| 2 | steA-KO-RP | TCAGTTTCTACCTATGCCAGAGCTTTATCAGGAAATAAGCGTGTAGGCTGGAGCTGCTTC |
| 3 | steA-eGFP cloning-FP | TTTTTCTCGAGATGCCATATACATCAGTTTCTACC |
| 4 | steA-eGFP cloning-RP | TTTTTGGATCCATAATTGTCCAAATAGTTATGG |
| 5 | HU-Rtn4a-RT-FP | TCGGGCTCAGTGGATGAGA |
| 6 | HU-Rtn4a-RT-RP | GCAGGACAGATGGGAAATCCT |
| 7 | HU-Climp63-RT-FP | GCGTCGAGCAGAAGGTGC |
| 8 | HU-Climp63-RT-RP | CATGGATCCCATCCGAGAGG |
| 9 | Xho1_fwd_Native_promoter_steA_cloning | TTTTTCTCGAGCGGCAGTGATTGCGTTGC |
| 10 | HindIII steA-HA_cloning_RP | TTTTTTAAGCTTTTATGCATAATCCGGAACATCATACGGATAATAATTGTCCAAATAGTTATGGTAGCGAGC |
| 11 | HU-β-actin-FP | GCCGCCAGCTCACCAT |
| 12 | HU-β-actin-RP | TCGTCGCCCACATAGGAATC |
